# Insulin-Regulated Aminopeptidase Inhibition Ameliorates Metabolism in Obese Zucker Rats

**DOI:** 10.3389/fmolb.2020.586225

**Published:** 2020-12-04

**Authors:** Katarina Krskova, Lucia Balazova, Viktoria Dobrocsyova, Rafal Olszanecki, Maciej Suski, Siew Yeen Chai, Štefan Zorad

**Affiliations:** ^1^Institute of Experimental Endocrinology, Biomedical Research Center, Department of Endocrine Regulations and Psychofarmacology, Slovak Academy of Sciences, Bratislava, Slovakia; ^2^Department of Pharmacology, Jagiellonian University Medical College, Cracow, Poland; ^3^Monash Biomedicine Discovery Institute, Department of Physiology, Monash University, Clayton, VIC, Australia

**Keywords:** obesity, insulin resistance, insulin-regulated aminopeptidase, IRAP, HFI-419

## Abstract

The aim of our study was to determine the influence of inhibition of insulin-regulated aminopeptidase/oxytocinase (IRAP) on glucose tolerance and metabolism of skeletal muscle and visceral adipose tissue in obese Zucker rats. Obese Zucker rats administered with IRAP inhibitor–HFI-419 at a dose of 29 μg/100 g BW/day by osmotic minipumps implanted subcutaneously for 2 weeks. Two-hour intraperitoneal glucose tolerance test (ipGTT) was performed in fasting rats. Plasma oxytocin levels were measured by enzyme immunoassay after plasma extraction. In the musculus quadriceps and epididymal adipose tissue, the expression of factors affecting tissue oxidative status and metabolism was determined by real-time qPCR and/or Western blot analysys. The plasma and tissue enzymatic activities were determined by colorimetric or fluorometric method. Circulated oxytocin levels in obese animals strongly tended to increase after HFI-419 administration. This was accompanied by significantly improved glucose utilization during ipGTT and decreased area under the curve (AUC) for glucose. In skeletal muscle IRAP inhibitor treatment up-regulated enzymes of antioxidant defense system – superoxide dismutase 1 and 2 and improved insulin signal transduction pathway. HFI-419 increased skeletal muscle aminopeptidase A expression and activity and normalized its plasma levels in obese animals. In epididymal adipose tissue, gene expression of markers of inflammation and adipocyte hypertrophy was down-regulated in obese rats after HFI-419 treatment. Our results demonstrate that IRAP inhibition improves whole-body glucose tolerance in insulin-resistant Zucker fatty rats and that this metabolic effect of HFI-419 involves ameliorated redox balance in skeletal muscle.

## Introduction

Obesity comprises one of the key risk factors for the metabolic syndrome and contributes to the development of insulin resistance and subsequently to type two diabetes mellitus, atherogenic dyslipidemia and cardiovascular disease (Ritchie and Connell, 2007). The high fat intake is associated with oxidative stress and activation of pro-inflammatory transcription factors (Ritchie and Connell, 2007). One of the most deleterious effects of obesity is the deposition of lipids in non-adipose tissues and hyperlipidenia-induced reactive oxygen species production leading to mitochondrial dysfunction in insulin-responsive tissues which might promote inhibition of insulin action (Di Meo et al., 2017). The Zucker fatty rat represents a well-established model of human obesity and insulin resistance. Obesity in this animal model is a consequence of spontaneous mutation (fa) in the gene encoding the leptin receptor resulting in hyperphagia (Phillips et al., 1996).

Insulin-regulated aminopeptidase (IRAP; leucyl-cystinyl aminopeptidase; oxytocinase), encoded by a *Lnpep* gene, has a broad distribution including skeletal muscle and white adipose tissue (Chai et al., 2004). IRAP is predominantly present in cytosolic vesicles together with the glucose transporter (GLUT4), from where they translocate to the plasma membrane upon insulin stimulation. The IRAP is a membrane bound protein belonging to the M1 family of aminopeptidases and its substrates include vasopressin and oxytocin (Chai et al., 2004). IRAP has been proposed as the surrogate marker of insulin-regulated vesicular traffic along with GLUT4 since the presence of IRAP is required for maintaining normal insulin-dependent translocation as well as forming an insulin-responsive vesicular compartment at the plasma membrane (Gross et al., 2004).

It has been found out that obesity in Zucker rats and also ob/ob mice is associated with a high plasma aminopeptidase A (AP-A) activity, the enzyme responsible for generating angiotensin III, thus contributing to blood pressure control (Morais et al., 2017; Lory et al., 2019). In our previous study we have shown that increased AP-A release from the skeletal muscle in obese Zucker rats significantly contributes to elevated plasma AP-A activity (Lory et al., 2019).

Recently, we have shown that obesity is accompanied by marked reduction of plasma oxytocin level in Zucker fatty rats caused by increased peptide degradation by liver and adipose tissue (Gajdosechova et al., 2014). This study highlighted the importance of the oxytocin system in the pathogenesis of obesity and suggested oxytocinase inhibition to improve obesity-induced metabolic disturbances. Several studies have examined the role of IRAP in obesity and glucose handling using various animal models. Results have revealed that insulin regulated traffic of IRAP toward membrane fraction in adipocytes is disturbed by monosodium glutamate-induced obesity (Alponti et al., 2015) and IRAP deficiency in mice fed high-fat diet lead to prevention of development of obesity (Niwa et al., 2015). On the other hand, acute inhibition of IRAP aminopeptidase activity with specific inhibitor, HFI-419, does not affect glucose homeostasis in the streptozotocin-induced experimental rat model of diabetes mellitus (Albiston et al., 2017). However, the effect of inhibitor of aminopeptidase activity HFI-419 on the physiology of skeletal muscle and adipose tissue in obese pre-diabetic Zucker rats has not been examined yet. The main hypothesis of our study was that prolonged treatment with IRAP inhibitor moderates oxytocin degradation in tissues, normalizes plasma oxytocin level and alleviates the obese phenotype in Zucker rats.

The aim of our study was to investigate the impact of HFI-419 application on the (i) whole-body metabolic parameters, (ii) expression of the markers of oxidative stress in skeletal muscle and adipose tissue, and (iii) expression of the tissue-specific parameters involved in their (pato) physiology in obese Zucker rats.

## Methods

### Animals

Male Zucker fatty rats (fa/fa) were purchased from Harlan (Udine, Italy). The animals were housed in a 12-h light/dark cycle with access to water and standard diet *ad libitum*. At the age of 34 weeks, the animals were divided into three groups. The control group of lean (*n* = 6) as well as the control group of obese (*n* = 6) rats received vehicle (30% cyclodextrin solution) and the experimental group of obese rats (*n* = 6) received IRAP inhibitor (HFI-419) (Merck KGaA, Darmstadt, Germany) in a dose 29 μg/100 g body weight/day dissolved in 30% solution of cyclodextrin) for two weeks via osmotic minipumps (ALZET, CA, United States) implanted subcutaneously, as described previously (Eckertova et al., 2011). After the minipumps were implanted, the rats were housed two per cage and were separated by a transparent barrier. On the 12th day, animals were subjected to intraperitoneal glucose tolerance test (ipGTT). After 2 days of recovery, overnight-fasted animals were sacrificed by decapitation at the age of 34 weeks. Experimental procedures involving animals were approved by the Jagiellonian University Ethical Committee on Animal Experiments and conformed to Declaration of Helsinki.

### Intraperitoneal Glucose Tolerance Test

The ipGTT was performed to assess glucose clearance. Overnight-fasted rats were administered an intraperitoneal injection of 50% glucose (w/v) at a dose of 2 g/kg body weight. The blood glucose was measured in the tail vein blood prior to and 30, 60, 90, and 120 min after glucose administration using a glucometer (Accu-Check Active, Roche Diagnostics, Switzerland).

### Measurement of Selected Metabolic Parameters and Hormones

After decapitation the trunk blood was collected in cooled tubes containing EDTA as anticoagulant and centrifuged immediately at 4°C to separate plasma, which was stored in aliquots at −20°C until analyaed. Fasting plasma insulin level was measured using commercial radioimmunoassay kit (Millipore, Bedford, MA, United States) following the manufacturer’s protocol. Fasting plasma glucose levels were measured using the multianalyser COBAS Integra 800 (Roche Diagnostics Ltd., Rotkreuz, Switzerland). Plasma lipid profile determination was performed using commercially available kits (Roche Molecular Diagnostics, Pleasenton, CA, United States). Quantitative insulin sensitivity check index (QUICKI) was calculated as follows: inverse of the sum of the logarithms of the fasting insulin (μU/ml) and fasting glucose (mg/dl). Plasma oxytocin concentrations were measured by EIA (Phoenix Pharmaceuticals, Burlingame, CA, United States) after extraction of the peptides using C-18 SEP COLUMN, following the manufacturer’s instructions. Precision of the assay declared by the manufacturer is: intra-assay variation <10%; inter-assay variation: <15%. Plasma C-peptide 2 concentrations were measured by ELISA (EMD Millipore Corporation, St. Louis, MO, United States) following the manufacturer’s protocol.

### RNA Isolation and Real-Time PCR

Prior to sampling for RNA, dissected samples from *musculus quadriceps* and epididymal adipose tissues were removed, frozen in liquid nitrogen and stored at –80°C until analysis. Total RNA was isolated using RNeasy Plus Universal Mini Kit (Qiagen, Valencia, CA, United States) following the manufacturer’s instructions. The reverse transcription of isolated RNA was performed using Maxima First Strand cDNA Synthesis Kit (Thermo Fisher, Waltham, MA, United States) according to the manufacturer’s protocol. Real-time PCRs were carried out applying Maxima SYBR Green qPCR Master Mix (Thermo Fisher, Waltham, MA, United States) and run on an ABI 7900HT thermal cycler (Applied Biosystems, Life Technologies, Carlsbad, CA, United States) using rat-specific primer pairs as described previously (Dobrocsyova et al., 2020). Data were normalized to the expression of housekeeping gene ribosomal protein S29 (*Rps29*) which was not altered by the treatment.

### Measurement of Enzyme Activity

Activity of glutamyl aminopeptidase was determined in the membrane fraction. Skeletal muscle and epididymal adipose tissue were homogenized using a glass Teflon homogenizer in lysis buffer (250 mM saccharose, 10 mM Tris, pH 7.4). The homogenates were centrifuged at 1,000 × *g*/10 min/4°C. The supernatants were collected and centrifuged at 16,000 × *g*/15 min/4°C to separate the membrane fraction. Protein concentration was measured by the Bicinchoninic Acid Protein Assay (Sigma-Aldrich, St. Louis, MO, United States). Prepared samples were mixed with substrate solution containing 100mM H-Glu-β-naphthylamide (Bachem, Bubendorf, Switzerland), 10 mg/100 ml bovine serum albumin, 10 mg/100 ml dithiothreitol, 50 mM CaCl2 in 50 mM Tris pH 7.4. The 96-well plate was placed in a Synergy H4 Hybrid Reader (BioTek, Winooski, VT, United States) fluorimeter and the enzyme kinetics was measured during 60 min at 37°C with data collection in 5-min intervals as the amount of β-naphthylamide released from the substrate due to the enzyme activity of AP-A at wavelengths 340 nm (excitation) and 410 nm (emission).

Superoxide dismutase activity was measured in skeletal muscle tissue samples by colorimetric SOD Activity Assay Kit (Abcam, Cambridge, United Kingdom). Tissue homogenization and assay procedure were performed in accordance with the manufacturer’ protocol.

### Western Blot

Quadriceps muscle was homogenized as described previously (Gajdosechova et al., 2014). After separation of proteins by SDS-PAGE electrophoresis and theirs transfer to a PVDF membrane (Immobilon-FL, Millipore, Bedford, MA, United States) the blots were incubated with primary antibody overnight at 4°C against insulin receptor substrate 1 (IRS-1) total protein or its phosphorylated form at residue Ser307 and Ser612 (#2382, #2381, and #2386, respectively; all purchased in Cell Signaling Technology, Danvers, MA, United States), insulin receptor β (IRβ) total and phosphorylated IRβ at Tyr1150/1151 residue (#3025 and #3024, Cell Signaling Technology, Danvers, MA, United States), SOD1, SOD2, NAD-dependent protein deacetylase sirtuin-1 (SirT1) and GAPDH (#37385, #13141, #9475, and #5174, respectively; Cell Signaling Technology, Danvers, MA) diluted (1:1000) in blocking buffer containing 0.1% Igepal. After membrane washing, the signal of fluorescently labeled secondary anti-rabbit IgG (#5151; Cell Signaling Technology, Danvers, MA, United States) was detected using the Odyssey infrared imaging system (LI-COR Biosciences, Lincoln, NE, United States) and quantified by Odyssey IR imaging system software version 2.0.

### Statistical Analysis

The results are presented as mean ± SEM. Analysis of normally distributed data was performed using the Kolmogorov-Smirnov test. Non-normally distributed data were subjected to natural logarithm transformation prior to statistical analysis. ANOVA with repeated measures was used to analyze glycemia at different time points of glucose tolerance test. Differences in basic metabolic and morphometric parameters, hormone levels and total area under the curve (AUC) for the glucose between experimental groups were analyzed by one-way ANOVA and differences between obese rats treated with vehicle and obese rats treated with HFI-419 aimed at determing skeletal muscle and adipose tissue metabolism were analyzed using Student’s *t*-test. Overall level of statistical significance was reached at ^∗^*p* < 0.05, ^∗∗^*p* < 0.01, and ^∗∗∗^*p* < 0.001.

## Results

We determined the basic characteristics of systemic metabolic and morphometric parameters in lean and obese Zucker rats and obese Zucker rats treated with HFI-419 for two weeks. As expected, obesity in Zucker (*fa/fa*) rats was accompanied by an increase in plasma insulin, C-peptide 2, triglycerides, cholesterol and LDL/HDL ratio, decreased insulin sensitivity index (QUICKI) and plasma oxytocin level without significant changes in fasting glycemia (Table 1). Plasma oxytocin concentration displayed a strong tendency (*p* = 0.058) toward increase after HFI-419 treatment. Two weeks administration of HFI-419 had no statistically significant effect on obesity-induced impaired metabolic and morphometric parameters (Table 1).

**TABLE 1:** Metabolic parameters in lean and obese Zucker rats treated with vehicle and obese Zucker rats treated with HFI-419.

	**leanVEH (*n* = 6)**	**obVEH (*n* = 6)**	**obHFI-419 (*n* = 6)**	***ANOVA p***
Body weight (g)	419.2 ± 18.0	608.5 ± 33.3 ***	161.0 ± 9.5 ***	<0.001
Fasting glycemia (mmol/l)	6.30 ± 0.20	6.48 ± 0.10	6.63 ± 0.27	0.523
Fasting insulin (ng/ml)	0.93 ± 0.20	10.91 ± 1.07 ***	11.66 ± 0.79 ***	<0.001
C-peptide (pM)	266 ± 37	1745 ± 121 ***	1734 ± 87 ***	<0.001
QUICKI^#^	0.294 ± 0.010	0.220 ± 0.002 ***	0.218 ± 0.002 ***	<0.001
2-h glycemia (mmol/l)	5.97 ± 0.44	9.75 ± 0.61 ***	8.33 ± 0.30 ***	0.001
Triglycerides (mmol/l)	1.07 ± 0.07	3.54 ± 0.57 ***	4.21 ± 0.69 ***	<0.001
Cholesterol (mmol/l)	2.48 ± 0.09	6.40 ± 0.41 ***	7.18 ± 0.70 ***	<0.001
LDL/HDL	0.205 ± 0.015	0.418 ± 0.037 *	0.510 ± 0.108 *	0.005
Plasma oxytocin (pg/ml)	26.98 ± 8.50	2.88 ± 1.01 **	7.79 ± 1.74	0.013

^#^QUICKI: quantitative insulin sensitivity check index. Data are presented as mean ± SEM and were analyzed using one-way ANOVA with Holm-Sidak *post hoc* test: *p < 0.05, **p < 0.01, ***p < 0.001 vs. leanVEH.

**FIGURE 1:**
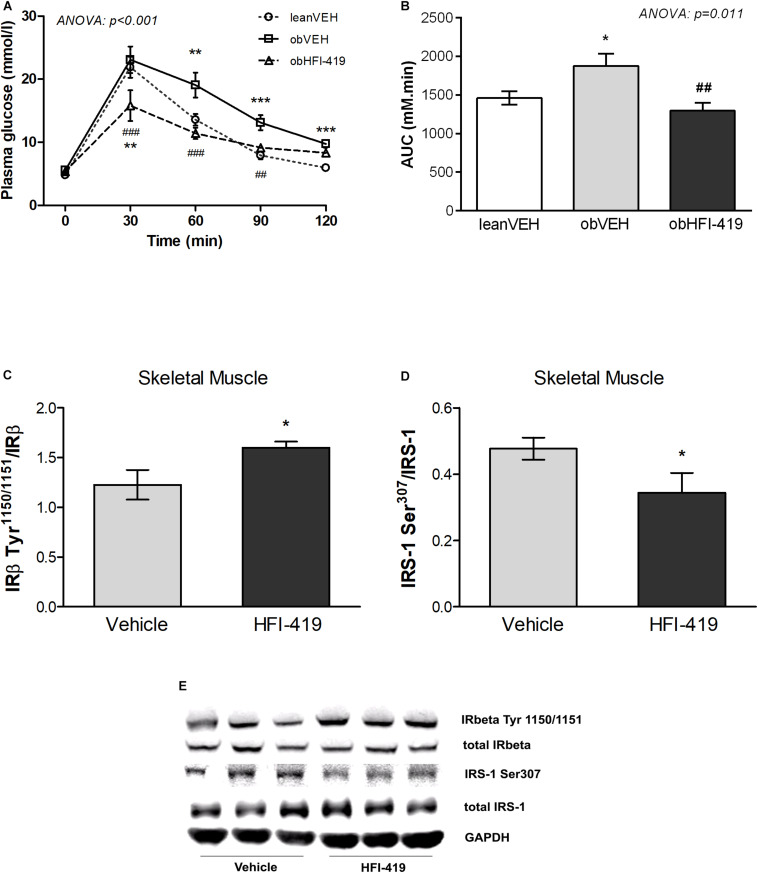
The effect of HFI-419 treatment on glucose utilization evaluated by intraperitoneal glucose tolerance test (ipGTT) in Zucker rats presented as absolute glucose concentrations **(A)** and as area under the curve (AUC) **(B)**. Data are shown as mean ± SEM and differences between experimental groups (*n* = 6) were analyzed by ANOVA with repeated measurements (for glucose concentrations) and one-way ANOVA (for AUC) with subsequent Holm-Sidak multiple comparison: *p < 0.05; **p < 0.01; ***p < 0.001 vs. lean vehicle group and ##*p* < 0.01; ###*p* < 0.001 vs obese vehicle group. The effect of HFI-419 treatment on phosphorylation of IRβ subunit at residue Tyr1150/1151 **(C)** and IRS-1 at residue Ser307 **(D)** in skeletel muscle determined as ratio between phosphorylated and total protein forms. Expressions of these proteins were evaluated by western blot and representative blots are shown at the panel **(E)**. Results are presented as mean ± SEM and differences between experimental groups (*n* = 6) were analyzed by Student’s *t*-test. *p < 0.05.

In order to determine the systemic effect of HFI-419 on glucose utilization, we performed ipGTT (Figure 1). The ANOVA with repeated measures revealed significant interaction between time and group of rats (*p* < 0.001) and subsequent *post hoc* analysis detected that HFI-419 treatment in obese rats led to decline of blood glucose at 30 (*p* < 0.001), 60 (*p* < 0.001), and 90 (*p* < 0.01) min after the glucose load when compared with obese animals treated with vehicle (Figure 1A). Similar marked tendency for 2-h glycemia to decrease (p = 0.065) was observed after the treatment (Table 1). Calculated AUC for glucose from ipGTT data was significantly reduced after HFI-419 administration (Figure 1B).

Regarding the insulin signaling pathway the protein expression of total insulin receptor β subunit (IRβ) and insulin receptor substrate (IRS-1) as well as phosphorylation of IRS-1 at residue Ser307 and Ser612 and IRβ at residue Tyr1150/1151 were evaluated in skeletal muscle. We did not observe statistically significant differences in total IRβ content (IRβ/GAPDH: Vehicle – 1.224 ± 0.092 vs. HFI-419 – 1.215 ± 0.127 a.u., *p* = 0.589) and total IRS-1 protein level (IRS-1/GAPDH: Vehicle – 0.553 ± 0.084 vs. HFI-419 – 0.499 ± 0.063 a.u. *p* = 0.621) after HFI-419 administration. The ratios of phospho-IRβ (Tyr1150/1151)/IRβ was significantly increased by HFI-419 treatment while the phospho-IRS-1 (Ser307)/IRS-1 ratio was significantly reduced by IRAP inhibitor application (Figures 1C,D). The ratio of phospho-IRS-1 (Ser612)/IRS-1 was unaffected by the treatment (Ser612)/IRS-1: Vehicle – 0.238 ± 0.039 vs. HFI-419 – 0.252 ± 0.030 a.u. *p* = 0.780).

**FIGURE 2:**
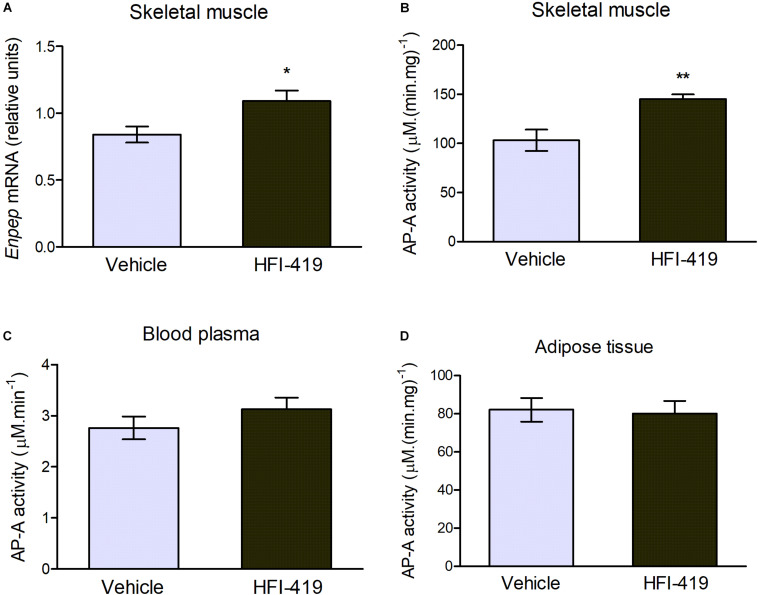
Aminopeptidase A (AP-A) activity and expression in peripheral tissues and plasma. **(A)** Gene expression of AP-A (coded by *Enpep* gene) in musculus quadriceps determined by real-time PCR. Data were normalized to the housekeeping gene encoding 40S ribosomal protein S29 (*Rps29*) whose expression was not significantly changed by the treatment. **(C)** AP-A activity in the plasma and in the membrane fractions isolated from **(B)** skeletal muscle and **(D)** epididymal adipose tissue. Data are expressed as mean ± SEM Differences between vehicle – (*n* = 6) and HFI-419 – treated (*n* = 6) obese Zucker rats were analyzed by Student’s *t*-test: *p < 0.05; **p < 0.01.

The observed changes led us to an extensive investigation of impact of HFI-419 treatment on insulin-responsive tissues metabolism in obese Zucker rats. Regarding skeletal muscle glutamyl aminopeptidase (AP-A), which is disregulated in obesity, both *Enpep* mRNA level (Figure 1A) and AP-A activity (Figure 2B) were significantly upregulated in this tissue in obese Zucker rats after the treatment. AP-A activity in plasma (Figure 2C) as well as epididymal adipose tissue (Figure 2D) was not affected by HFI-419 administration.

**FIGURE 3:**
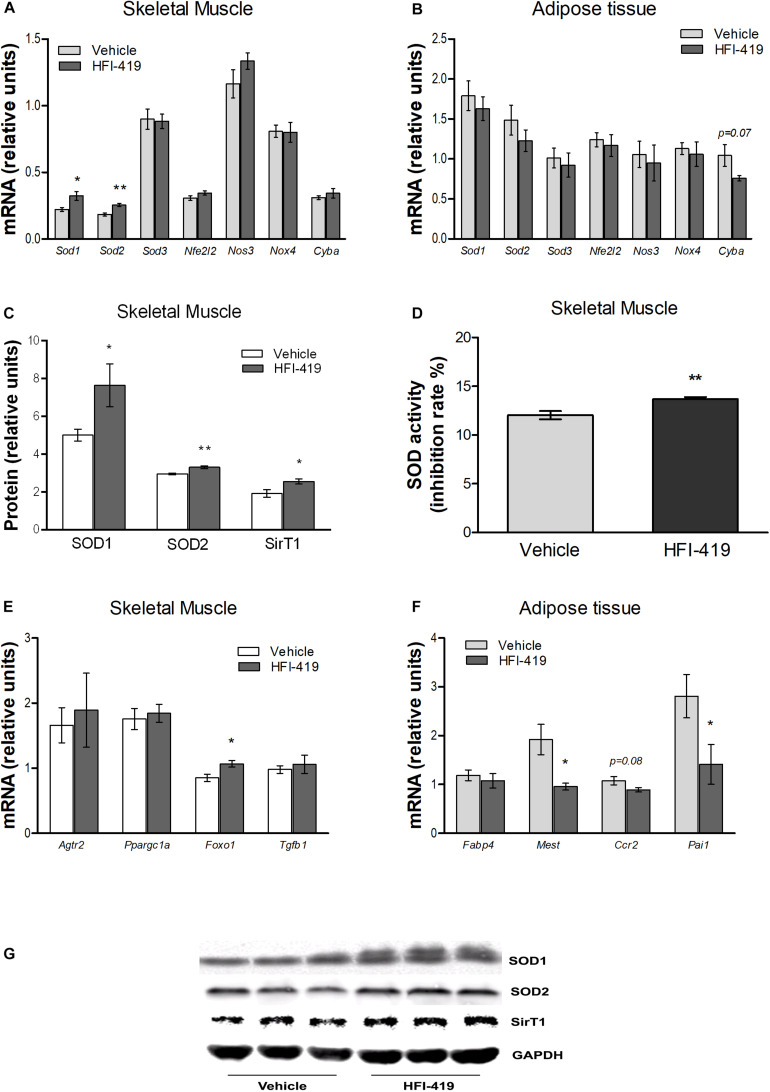
Effect of HFI-419 treatment on expression of genes and proteins with pro-oxidant and antioxidant action and genes coding proteins involved in skeletal muscle and adipose tissue metabolism. Gene expression of superoxide dismutase [Cu-Zn] (*Sod1*), superoxide dismutase [Mn] (*Sod2*), extracellular superoxide dismutase [Cu-Zn] (*Sod3*), nuclear factor erythroid 2-related factor 2 (*Nfe2l2*), endothelial nitric oxid syntase (*Nos3*), NADPH oxidase (*Nox4*) and cytochrome b-245 light chain (*Cyba*), type-2 angiotensin II receptor (*Agtr2*), peroxisome proliferator-activated receptor gamma coactivator 1-α (*Ppargc1a*), forkhead box protein O1 (*Foxo1*), transforming growth factor β-1 (*Tgfb1*), fatty acid-binding protein 4 (*Fabp4*), mesoderm-specific transcript homolog protein (*Mest*), C-C chemokine receptor type 2 (*Ccr2*) and plasminogen activator inhibitor 1 (*Pai1*) in musculus quadriceps **(A,E)** and epididymal adipose tissue **(B,F)** of vehicle– and HFI-419–treated obese Zucker rats determined by real-time PCR. Data were normalized to the gene expression of 40S ribosomal protein S29 (*Rps29*) whose expression was not altered by the treatment. Protein expression of SOD1 and SOD2 and silent information regulator 1 (SirT1) **(C)** and SOD activity **(D)** in skeletal muscle of obese vehicle– and HFI-419–treated rats. Expression of proteins was evaluated by western blot and obtained data were normalized to loading control GAPDH whose expression was not altered by the treatment. Representative blots are shown at the panel **(G)**. SOD activity is expressed as percentage of inhibition of tetrazolium salt WST-1 conversion to formazan dye and is related to 1 mg of tissue. Data are presented as mean ± SEM and were analyzed by Student’s *t*-test: *p < 0.05; **p < 0.01.

Since hyperlipidemia-induced ROS production in skeletal muscle is involved in inhibition of insulin action, we evaluated the mRNA level of markers of oxidative stress and antioxidant defence mechanism in this tissue as well as in epididymal adipose tissue. In skeletal muscle, statistically significant upregulation of *Sod1* and *Sod2* mRNA levels were detected after HFI-419 administration (Figure 3A). A similar stimulating effect of HFI-419 was found in the case of SOD1 and SOD2 protein expression and SOD enzyme activity (Figures 3C,D). The expression of other genes encoding enzymes with antioxidant properties (*Sod3, Nfe2l2*, and *Nos3*) and NOX4 and P22PHOX subunit of NADPH oxidase (*Nox4* and *Cyba*) were not significantly affected by the treatment. In adipose tissue, the expression of prooxidant and antioxidant genes was found unchanged except for *Cyba* mRNA level, which tended to decrease (*p* = 0.07) in rats treated with HFI-419 (Figure 3B).

In skeletal muscle, significantly elevated level of transcription factor forkhead box protein O1 (*Foxo1*) mRNA, involved in regulation of redox balance, were detected after HFI-419 treatment (Figure 3E). Interestingly, increased protein content of SirT1, regulator of *Foxo* transcriptional activity, were observed in skeletal muscle after HFI-419 administration (Figure 3C). The gene expressions of transforming growth factor β-1 (*Tgfb*1), peroxisome proliferator-activated receptor gamma coactivator 1-α (*Ppargc1a*), involved in mitochondrial biogenesis, and type-2 angiotensin II receptor (*Agtr2*) were not changed in obese Zucker rats receiving HFI-419 (Figure 3E).

Regarding the adipose tissue metabolism, the effect of HFI-419 treatment on the gene expression of markers involved in adipogenesis and inflammation were detected in epididymal fat depot of obese Zucker rats. The *Fabp4* mRNA level was not changed while mesoderm-specific transcript homolog protein (*Mest*) and plasminogen activator inhibitor 1 (*Pai1*) mRNA levels were significantly reduced and C-C chemokine receptor type 2 (*Ccr2*) transcript level tended to decrease (*p* = 0.08) in HFI-419–treated rats (Figure 3F).

## Discussion

The genetically obese Zucker rats (fa/fa) represent a model of insulin resistance and prediabetes characterized by hyperinsulinemia, hyperlipidemia and peripheral glucose intolerance which is caused largely by an impairment of insulin-stimulated glucose uptake into skeletal muscle, the major site of insulin-mediated glucose disposal (Ionescu et al., 1985; King et al., 1992). We and other authors have previously shown that obesity is associated with dysregulation of IRAP activity in adipose tissue and skeletal muscle along with reduced plasma oxytocin due to increased peptide degradation by peripheral tissues (Gajdosechova et al., 2014; Alponti et al., 2015). A study on IRAP-knockout mice showed protective effect of aminopeptidase deficiency against development of obesity (Niwa et al., 2015). In order to investigate the impact of IRAP inhibition on peripheral glucose tolerance and skeletal muscle and adipose tissue metabolism, we treated obese Zucker rats with HFI-419, a specific inhibitor of IRAP catalytic activity, for 2 weeks.

In our study, we confirmed reduced plasma oxytocin levels in obese eight-month-old rats compared with lean animals. The inhibition of aminopeptidase activity by HFI-419 resulted in a strong elevating tendency of circulating oxytocin level which was accompanied with marked amelioration of the glucose utilization during ipGTT in obese rats treated with IRAP inhibitor. Based on decrease of AUC and 2-h glycemia (*p* = 0.065) to control values in HFI-419 – treated rats, we assume that inhibition of IRAP substantially improved glucose tolerance in obese rats. Decreased blood glucose level at 30 min in obese treated rats compared to both untreated obese and control lean rats during ipGTT may indicate that the HFI-419 treatment could enhance the insulin secretion after the glucose injection. However, plasma insulin and C-peptide was not determined during ipGTT in our study. Regarding peripheral glucose tolerance, our results are consistent with previous observations in IRAP-deficient mice (*Irap-/-*) in which glucose and insulin tolerance tests have revealed that the glucose disposal and the hypoglycemic effect of insulin did not differ between groups of animals fed a normal diet but both of those are improved in *Irap-/-* mice after a high-fat diet challenge (Niwa et al., 2015). On the contrary, acute single administration of IRAP inhibitor HFI-419 did not affect peripheral whole-body glucose handling during the glucose and insulin tolerance tests, neither in control rats nor in the streptozotocin-induced experimental rat model of diabetes mellitus type I (Albiston et al., 2017). In keeping with these results, IRAP *in vitro* inhibition (30-min and 24-h) of both basal and insulin-stimulated L6GLUT4myc cells did not alter glucose uptake (Albiston et al., 2017). Thus, the effect of HFI-419 on glucose utilization seems to depend on metabolic status of the animals with the positive effect seen only in obese and insulin resistant ones.

With regard to potential mechanism for improvement of glucose utilization in HFI-419–treated animals we found beneficial changes in the insulin signaling cascade in the skeletal muscle. The ratio of phospho-IRβ(Tyr1150/1151)/IRβ was increased after HFI-419 administration. Insulin receptor subunit β phosphorylation in the Tyr11501151 has been demonstrated to be an important control site for transmission of the insulin signal (White et al., 1988). Next we found significantly decreased the ratio of phospho-IRS-1(Ser307)/IRS-1 and unaffected phospho-IRS-1(Ser612)/IRS-1 ratio in skeletal muscle of HFI-419–treated rats. Increased phosphorylation of IRS-1 on residues Ser307 and Ser612 have been suggested to be responsible for the desensitization mechanisms of insulin-stimulated signal transduction which plays an important role in insulin resistance in obesity (Aguirre et al., 2000). Thus, our results suggest that IRAP inhibition has a beneficial effect on glucose metabolism in the skeletal muscle.

We have recently detected obesity-associated significant decrease of membrane-bound AP-A (glutamyl aminopeptidase) activity in the skeletal muscle of Zucker rats accompanied by elevated plasma AP-A activity suggesting stimulated tissue AP-A autolysis due to dyslipidemia in obese phenotype (Lory et al., 2019). AP-A hydrolyses angiotensin II to the heptapeptide angiotensin III which has the highest relative affinity for AT2 receptor and is the most potent endogenous AT2 receptor agonist (Bosnyak et al., 2011). After HFI-419 treatment, we found a significant increase of AP-A gene expression along with a significant increase of membrane-bound AP-A activity in skeletal muscle. Such an upregulation of tissue AP-A activity was not observed in the plasma implicating that IRAP inhibitor has the potential to normalize obesity-impaired release of AP-A into the circulation and normalize the levels of its soluble form. In our previous study, we found a significant (10-fold) increase in AT2 receptor and a parallel decrease in AT1 receptor gene expression in obese 8-month-old Zucker rats (Lory et al., 2019). Regarding skeletal muscle physiology, AT2 receptor activation improves skeletal muscle perfusion, glucose uptake and oxygenation and is required for normal muscle microvascular and metabolic responses to insulin (Chai et al., 2011).

In relation to hyperlipidemia-induced ROS production and mitochondrial dysfunction associated with development of insulin resistance in skeletal muscle, we evaluated expression of genes encoding proteins with pro- and antioxidant properties and found out that HFI-419 treatment elevates the *Sod1* and *Sod2* mRNA levels while the expression of prooxidative genes (*Nox4* and *Cyba*) did not change. Increased superoxide dismutases gene expressions were also reflected in an increase in the SOD1 and SOD2 protein levels as well as in an increase in the SOD enzyme activity in the treated animals. The first line of antioxidant defense systems, critical in maintaining cellular redox balance, consists of superoxide dismutases (SODs), which eliminates superoxide to produce the less reactive H_2_O_2_: the cytosolic Cu/Zn-containing SOD (CuZnSOD encoded by *Sod1* gene), the mitochondrial Mn-containing SOD (MnSOD encoded by *Sod2* gene), and the extracellular SOD (EcSOD encoded by *Sod3* gene) (Di Meo et al., 2017). Several lines of evidence have been provided that *Sod1* overexpression improves insulin-dependent glucose uptake into cells as well as peripheral glucose tolerance (Hoehn et al., 2009; Boden et al., 2012). Furthermore, *MnSOD* −/+mice displayed a significant impairment in glucose tolerance despite similar insulin levels to control mice (Hoehn et al., 2009). This upregulation of *Sod1* and *Sod2* genes observed in our study indicate that HFI-419 treatment has a beneficial effect on the skeletal muscle redox homeostasis, which is reflected by the improved glucose utilization. The up-regulation of antioxidant enzymes SOD1 and SOD2 observed in our study suggests that HFI-419 treatment has an improving effect on the skeletal muscle redox homeostasis, which might contribute to skeletal muscle insulin sensitivity.

A novel observation of the present study is that IRAP inhibition induced an elevation of *Foxo1* mRNA, transcription factor highly expressed in the major insulin target tissues, as well as protein content of SirT1, regulator of FoxO transcriptional activity in skeletal muscle of obese rats. It has been shown, that under pathophysiological conditions such insulin resistance and metabolic dysfunction FoxO1 expression may help drive the expression of genes involved in combating oxidative stress and that there is interplay between SirT1 and FoxO in ROS reduction. SirT1 also directly interacts with SOD, which could be regulated through FoxO (Gross et al., 2008; Zhang et al., 2017). It is tempting to speculate, that HFI-419–induced up-regulation of SirT1 protein may be involved in subsequent SOD upregulation and amelioration of oxidative stress in skeletal muscle of obese Zucker rats.

In relation to adipose tissue metabolism, plasminogen activator inhibitor 1 (PAI-1), a key component of fibrinolysis that has been demonstrated within adipose tissue being involved in adipose tissue expansion and insulin resistance was found to be reduced at the mRNA level after treatment with HFI-419. Study using *Irap-/-* mice revealed that IRAP deficiency may lead to attenuated PAI-1 expression in differentiated preadipocytes isolated from inguinal fat pads of mice, since adipocyte *Pai1* mRNA as well as PAI-1 protein and its secretion into the culture medium were lower in adipocytes from *Irap-/-* mice than those of wild-type mice (Wang et al., 2018). PAI-1 also contributes to the development of inflammation in adipose tissue during obesity by mechanism involving up-regulation of M1 (clasically activated) macrophage numbers in visceral adipose depot (Wang et al., 2018). In addition to down-regulated *Pai1* mRNA in our study, the gene expression of C-C chemokine receptor type 2 (*Ccr2*), a marker of adipose tissue macrophage infiltration and accumulation (Kim et al., 2016), and mesoderm-specific transcript homolog protein (*Mest*), displayed clear tendency to decline by HFI-419 application. MEST is thought to play a role in the facilitation of lipid accumulation and fat storage in adipocytes, adipogenesis and adipocyte hypertrophy (Takahashi et al., 2005; Prudovsky et al., 2018). Our study confirmed *in vivo* previously observed *in vitro* changes in PAI-1 and has showed a role for IRAP in the regulation of these adipocyte markers.

In summary, our results show that the specific inhibitor of aminopeptidase activity of IRAP, HFI-419, ameliorated the obesity-induced metabolic disturbances in in obese Zucker rats. Beneficial effect was manifested by improvement of the peripheral whole-body glucose tolerance as well as activation of skeletal muscle antioxidant mechanisms and insulin signaling pathway. The results presented are likely to motivate further research as there is a gap in the knowledge regarding the regulation of IRAP/oxytocinase in order to reveal new approaches to modulate metabolism in obesity.

## Data Availability Statement

The raw data supporting the conclusions of this article will be made available by the authors, without undue reservation.

## Ethics Statement

The animal study was reviewed and approved by the Jagiellonian University Ethical Committee on Animal Experiments.

## Author Contributions

KK contributed to the study with the acquisition, analysis, and interpretation of the data, and wrote the first manuscript draft. LB contributed to the conception and design of the study, and participated in the performance of experiments and acquisition of data. VD participated in the performance of animal experiments and acquisition of data. RO contributed to the conception and design of the study and supervised the experiments with animals. MS contributed to the conception and design of the study and participated in the performance of animal experiments. SC contributed to the conception and design of the study and participated in the methodology. ŠZ participated in the performance of experiments, supervised the research, contributed with the project administration, and provided critical revision of the final form of the manuscript. All authors contributed to the article and approved the submitted version.

## Conflict of Interest

The authors declare that the research was conducted in the absence of any commercial or financial relationships that could be construed as a potential conflict of interest.
